# AI tool use, self-efficacy, foreign language enjoyment, and willingness to communicate: a moderated serial mediation model among Chinese EFL learners

**DOI:** 10.3389/fpsyg.2026.1829287

**Published:** 2026-07-21

**Authors:** Linshen Xu, Yiming Wang, Yixuan Feng

**Affiliations:** 1 School of Foreign Studies, Hebei Normal University, Shijiazhuang, China; 2 Department of Aeronautical Engineering, Shijiazhuang Engineering Vocational College, Shijiazhuang, China

**Keywords:** AI self-efficacy, AI tool use, Chinese EFL learners, foreign language anxiety, foreign language enjoyment, moderated serial mediation, willingness to communicate

## Abstract

**Introduction:**

The rapid proliferation of artificial intelligence (AI) tools in higher education has transformed the landscape of foreign language instruction, yet the mechanisms through which AI tool use shapes learners' affective and communicative engagement remain poorly understood. Drawing on social cognitive theory (SCT) and control-value theory (CVT), this study proposes and tests a moderated serial mediation model in which AI tool use predicts willingness to communicate (WTC) sequentially through AI self-efficacy and foreign language enjoyment (FLE), with foreign language anxiety (FLA) moderating the FLE-to-WTC link.

**Methods:**

Survey data were collected from 420 EFL undergraduates at two Chinese universities (Zhejiang and Shaanxi Provinces). Covariance-based structural equation modeling (CBSEM) with maximum likelihood robust (MLR) estimation in Mplus 8.8 was used.

**Results:**

The model revealed excellent fit. AI tool use significantly predicted AI self-efficacy (β = 0.42, *p* < 0.001), which predicted FLE (β = 0.38, *p* < 0.001) and WTC directly (β = 0.21, *p* = 0.001); FLE predicted WTC (β = 0.33, *p* < 0.001). Bootstrapped mediation analyses (10,000 replications) confirmed a simple indirect effect via self-efficacy [β = 0.091, 95% CI (0.042, 0.152)] and a serial indirect effect via self-efficacy and enjoyment [β = 0.053, 95% CI (0.021, 0.098)]. FLA moderated the FLE-to-WTC pathway (β = −0.19, *p* = 0.002), and the index of moderated mediation was significant [IMM = −0.017, 95% CI (−0.038, −0.004)].

**Discussion:**

These findings advance theoretical understanding of how AI tools cultivate positive affective trajectories and carry implications for emotionally responsive EFL pedagogy.

## 1 Introduction

Consider a first-year university student in Lanzhou who, the night before an oral examination, turns not to a textbook but to a conversational AI assistant. She rehearses answers, receives instant corrective feedback, and gradually feels something unexpected: the fear that had paralyzed her for three semesters begins to recede, replaced by a cautious confidence that she might, after all, have something worth saying in English. Whether this anecdotal shift—from AI-assisted practice to genuine willingness to speak—reflects a systematic psychological process is the central question motivating the present research.

AI-powered tools—including large language model chatbots, intelligent grammar checkers, and automated pronunciation coaches—have entered mainstream EFL classrooms with remarkable speed. Surveys of Chinese university students consistently report that the majority use at least one AI tool for English study, with ChatGPT-type applications and AI translation assistants among the most common (Balci, 2024; Çobanogullari and Özbek, 2025). Yet adoption statistics tell us little about whether, and through what mechanisms, AI tool use translates into meaningful communicative engagement. Willingness to communicate (WTC), defined as the learner's readiness to initiate second-language (L2) communication given the choice (MacIntyre et al., 1998), is widely regarded as a proximal determinant of actual language use and long-term proficiency growth. Understanding how AI tool use is linked to WTC is therefore both theoretically important and practically urgent. Unlike research grounded in the technology acceptance model (TAM) or the unified theory of acceptance and use of technology (UTAUT), which seek to explain the antecedents of technology adoption, the present study does not examine acceptance *per se*; instead, it asks how AI tool use shapes communicative behavior through intervening affective mechanisms. Accordingly, two research questions guide the study. RQ1: Does AI tool use predict WTC through sequential mediation via AI self-efficacy and foreign language enjoyment? RQ2: Does foreign language anxiety moderate the enjoyment-to-WTC pathway such that the beneficial effect of enjoyment is weaker for high-anxiety learners?

Two theoretical frameworks guide the present investigation. Social cognitive theory (SCT; Bandura, 1997) posits that self-efficacy beliefs—individuals' confidence in their capacity to perform specific tasks—are critical mediators between environmental affordances and goal-directed behavior. In the L2 domain, learners who perceive AI tools as effective scaffolds are likely to develop stronger beliefs in their own language ability, which should, in turn, expand their willingness to engage communicatively. Alongside SCT, control-value theory (CVT; Pekrun, 2006) proposes that academic emotions—including enjoyment—are generated by learners' appraisals of control over and value of learning activities. Because AI tools may heighten both perceived control (instant feedback, adjustable difficulty) and value (authentic communicative practice), they are well positioned to elicit foreign language enjoyment (FLE). FLE, in turn, has been identified as a robust positive predictor of WTC (Dewaele and MacIntyre, 2014; Jiang and Dewaele, 2019). The present model therefore proposes a serial mediation pathway: AI Tool Use → AI Self-Efficacy → FLE → WTC.

However, this positive pathway may not be universally available. Foreign language anxiety (FLA)—a situation-specific apprehension about L2 use (Horwitz et al., 1986)—is pervasive among Chinese EFL learners and has been shown to impede communication even when learners report enjoyment (Horwitz, 2001; Khajavy et al., 2018). We therefore propose that FLA moderates the FLE-to-WTC link, attenuating the beneficial effect of enjoyment for anxious learners. This moderated serial mediation design integrates both affective enablers and inhibitors of L2 communication within a unified model, extending prior work that has examined these constructs separately.

Despite the proliferation of studies on AI in L2 education, three gaps remain. First, most research treats AI tool use as an undifferentiated independent variable, without articulating the psychological mechanisms through which technology shapes communication. Second, while both self-efficacy and FLE have been studied as mediators independently, their sequential relationship—in which self-efficacy precedes and predicts enjoyment—has rarely been tested in an AI context. Third, the boundary conditions of AI-driven enjoyment on WTC have received scant empirical attention. The present study addresses all three gaps by testing the full moderated serial mediation model with a large, geographically diverse sample of Chinese EFL undergraduates.

China provides an especially instructive context in which to examine these questions. With over 300 million EFL learners and a national policy environment that actively promotes AI integration into tertiary education, Chinese universities represent both the largest single EFL learner population and one of the most technology-intensive English learning ecosystems in the world. At the same time, Chinese EFL learners are characterized by exceptionally high FLA and comparatively low WTC, making them an important population for understanding how AI tools might—or might not—overcome long-standing psychological barriers to communicative engagement.

The remainder of this article reviews the theoretical background and develops eight hypotheses, describes the method, reports results, and discusses implications for theory and EFL pedagogy.

## 2 Theoretical framework and hypotheses

### 2.1 AI tool use in foreign language learning

Because willingness to communicate (WTC) is the focal outcome of the present model, we foreground it here before turning to its antecedents. WTC denotes a learner's readiness to enter into L2 communication when free to do so (MacIntyre et al., 1998); it is a proximal predictor of authentic language use and, in Chinese EFL contexts in particular, a recognized bottleneck in the transition from linguistic competence to actual communication. The sections that follow trace how AI tool use may feed into this outcome through a sequence of cognitive and affective antecedents, with a fuller treatment of the WTC construct provided in Section 2.5.

The term “AI tool use” in the present study refers to the frequency and variety with which EFL learners engage AI-powered applications—including conversational chatbots, intelligent writing assistants, AI-based translation tools, and automated feedback systems—in service of English learning. This operational definition encompasses both deliberate study-oriented use (e.g., practicing dialogue with a chatbot) and incidentally language-related use (e.g., using AI translation to check comprehension). The technological affordances of these tools—immediate, low-stakes feedback; personalized difficulty calibration; and always-available conversation partners—distinguish them qualitatively from prior computer-assisted language learning (CALL) technologies (Kasneci et al., 2023).

Empirical evidence on AI tool use in EFL contexts is accumulating rapidly. A systematic review by Balci (2024) identified positive effects of ChatGPT use on writing quality, speaking confidence, and vocabulary acquisition across tertiary EFL settings. Çobanogullari and Özbek (2025) developed and validated a dedicated scale for AI tool use in foreign language learning, finding that tool-use frequency was positively associated with self-reported language improvement. These findings are consistent with a technology acceptance perspective (Davis, 1989), which predicts that perceived usefulness and ease-of-use drive continued engagement with learning technologies. Yet neither study explicitly modeled the psychological intermediaries linking tool use to communicative outcomes.

The empirical landscape of AI in EFL education has expanded substantially in recent years, encompassing AI-assisted writing feedback (Barrot, 2023; Luo et al., 2025; Ma et al., 2026; Wiboolyasarin et al., 2024; Yeung, 2025), learner identity and academic writing contexts (Hu et al., 2025), and GenAI-integrated curriculum design (Chen et al., 2026; Chen and Pi, 2026; Zhao et al., 2025). Cross-cultural investigations document how Vietnamese, Turkish, and Indian EFL educators negotiate AI integration within their pedagogical frameworks (Fatalaki et al., 2025; Mutlu, 2025; Pham and Le, 2026; Sharma and Rai, 2026), while global reviews of GenAI in ELT emphasize the psychological dimensions of AI adoption (Boyd and Markowitz, 2026; Lee et al., 2025; Sánchez-Trujillo et al., 2025). Notwithstanding this growing evidence base, theoretically grounded models that explain how learner-level AI tool use translates into affective and communicative outcomes remain scarce, motivating the present investigation.

### 2.2 Social cognitive theory, AI self-efficacy, and the WTC model

Bandura's (1997) social cognitive theory positions self-efficacy as the primary cognitive mechanism through which environmental experiences are translated into behavior. Self-efficacy beliefs develop through four main sources: mastery experiences, vicarious learning, social persuasion, and physiological/affective states. AI tools are plausibly associated with all four: they provide structured mastery experiences through incremental practice tasks, offer vicarious models via model-generated output, supply affirmative or corrective feedback that functions as social persuasion, and reduce the physiological anxiety typically associated with high-stakes L2 interaction. In the L2 domain, higher self-efficacy has been consistently linked to greater effort, persistence, and communicative risk-taking (Graham, 2022; Raoofi et al., 2012).

We conceptualize “AI self-efficacy” as a domain-specific variant of general L2 self-efficacy: learners' confidence in their ability to use English effectively when supported by AI tools. This construct bridges technology acceptance (Davis, 1989) and language self-efficacy traditions. Learners who engage more frequently with AI tools should accumulate the mastery experiences and corrective feedback that build competence beliefs (Bandura, 1997). We therefore propose:

Following Bandura's (1997) principle of domain specificity—that efficacy beliefs are most predictive when assessed at the level of the domain in which they operate—AI self-efficacy is conceptually distinct from both general L2 self-efficacy and computer self-efficacy. Whereas general L2 self-efficacy concerns confidence in using the language across situations, and computer self-efficacy concerns confidence in operating digital technology irrespective of purpose, AI self-efficacy is defined more narrowly as learners' confidence in deploying AI tools to accomplish English-learning tasks. We acknowledge that confidence exercised in AI-mediated practice and broader communicative confidence are related; the construct is therefore operationalized with items that explicitly anchor the judgement to the AI-supported context (e.g., “When I use AI tools for English practice…”), and its discriminant validity relative to the other affective and cognitive constructs is examined empirically through the square root of the average variance extracted (AVE) and the heterotrait–monotrait (HTMT) ratio (see Section 4.2).

**H1:** AI tool use is positively associated with AI self-efficacy.

MacIntyre et al.'s (1998) heuristic model of WTC presents a layered pyramid of antecedents, with situational self-confidence—comprising both perceived competence and L2 anxiety—as the most proximal determinant of WTC at a given moment. Numerous studies have confirmed that self-efficacy, as an operationalization of perceived competence, is a direct positive predictor of WTC (Khajavy et al., 2018). We extend this logic to the AI-augmented context:

**H4:** AI self-efficacy is positively associated with WTC (direct path).

Furthermore, because AI self-efficacy should increase learners' readiness to use English beyond the safe AI interface—in actual communicative encounters—we propose a simple indirect path:

**H5:** AI tool use exerts a positive indirect effect on WTC through AI self-efficacy.

In the Chinese university EFL context, the cultivation of self-efficacy through AI tool use carries particular salience. Chinese EFL learners have historically reported lower L2 self-efficacy compared to counterparts in English-speaking or bilingual environments, a pattern attributed to face-saving cultural norms, an emphasis on accuracy over fluency in secondary school instruction, and limited authentic communicative practice opportunities (Khajavy et al., 2018; Li et al., 2020). AI tools disrupt this pattern by providing a private, non-judgmental interlocutor: the absence of peer observation removes the social evaluation threat that typically suppresses communicative risk-taking, allowing learners to accumulate mastery experiences without the anxiety cost normally associated with live L2 use. This “protected practice” affordance is theoretically central to the AI tool use → AI self-efficacy pathway proposed in H1.

It is also worth distinguishing AI self-efficacy from more general measures of L2 self-efficacy or computer self-efficacy (Davis, 1989). Whereas general L2 self-efficacy reflects beliefs about language competence broadly, AI self-efficacy specifically captures confidence in using English when AI-mediated scaffolding is available. This distinction matters empirically: a learner may feel confident practicing with an AI chatbot yet remain hesitant to initiate a conversation without technological support. The present study operationalizes AI self-efficacy to capture this affordance-specific competence belief, following the domain-specificity principle of Bandura's (1997) framework that self-efficacy beliefs are most predictive when assessed at the same level of specificity as the target behavior. Future research might examine whether AI self-efficacy generalizes to non-AI communicative contexts over time, and whether such generalization is moderated by the duration and variety of AI practice experience.

Recent instrument development work has advanced the precise measurement of AI-related competence beliefs in higher education. Xia et al. (2025) developed the Generative AI Competency Self-Efficacy Scale for Teachers and Researchers (GAICS-TR), confirming that domain-specific AI self-efficacy is empirically distinguishable from general technology self-efficacy. Similar domain-specificity principles have been demonstrated in L2 writing contexts: Tsao (2021) showed that self-efficacy assessed at the level of AI-assisted writing tasks predicted engagement with written corrective feedback more strongly than general L2 writing confidence. In the EFL development context, An and Li (2025) found that individual differences in confidence and self-regulatory capacity moderated the benefits of feedback conditions on L2 acquisition, while Lee (2022) similarly identified self-efficacy and affective variables as pivotal mediating factors in classroom-based L2 writing research.

### 2.3 Control-value theory and foreign language enjoyment

Pekrun's (2006) control-value theory (CVT) offers a comprehensive account of how learning environments generate academic emotions. The theory posits that learners' appraisals of control (ability to influence outcomes) and value (importance and interest of activities) determine whether activity-related emotions are positive (enjoyment, hope, pride) or negative (anxiety, boredom, hopelessness). Applied to the AI-EFL context, AI tools may increase both perceived control—by providing immediate, non-judgmental feedback that makes success more achievable—and perceived value—by enabling authentic communicative practice that learners find intrinsically interesting. CVT therefore predicts that higher AI self-efficacy (a proxy for control appraisal) should elicit greater FLE.

FLE, introduced as a construct by Dewaele and MacIntyre (2014), refers to the subjective experience of positive emotions—pleasure, amusement, excitement—specifically associated with using and learning a foreign language. Initial evidence using a 21-item FLE scale showed that FLE was empirically and conceptually distinct from its more extensively studied counterpart, foreign language classroom anxiety (FLCA; Horwitz et al., 1986). Subsequent studies have confirmed FLE's positive effects on participation, effort, and proficiency outcomes across a range of EFL contexts (Dewaele and Alfawzan, 2018; Dewaele et al., 2018; Li et al., 2020).

The present study proposes that AI self-efficacy, by signaling that learners are in control of AI-mediated language tasks, generates FLE in accordance with CVT appraisal mechanisms. We further propose that FLE is a distal driver of WTC: the positive affect associated with enjoyment broadens learners' cognitive and behavioral repertoires (Pekrun and Perry, 2014), making communicative engagement more intrinsically appealing. The mechanism linking competence beliefs to enjoyment is well attested empirically: control appraisals—a learner's sense of being in command of a task—are among the most consistent antecedents of positive activating emotions in the control-value framework (Pekrun, 2006), and self-efficacy has been shown to foster enjoyment and engagement in both technology-enhanced and language-learning settings (Derakhshan and Fathi, 2024; Han and Geng, 2023). The downstream link from enjoyment to communicative readiness is likewise empirically grounded: foreign language enjoyment predicts willingness to communicate across classroom and digital contexts (Lee, 2020; Lee and Chen Hsieh, 2019), consistent with a broaden-and-build account in which positive affect widens learners' behavioral repertoire and lowers the threshold for initiating L2 interaction. These theoretical arguments support the following hypotheses:

Although the FLE scale was originally developed to capture enjoyment experienced in the language classroom (Dewaele and MacIntyre, 2014), AI-supported study has become interwoven with both in-class and out-of-class English learning among Chinese university students, such that enjoyment arising from AI-mediated practice plausibly carries over into learners' overall enjoyment of learning the language (Lee and Chen Hsieh, 2019). We nonetheless treat the contextual span of the enjoyment measure as a matter warranting scrutiny and return to it in the Section 5.6.

**H2:** AI self-efficacy is positively associated with FLE.
**H3:** FLE is positively associated with WTC.
**H6:** AI tool use exerts a positive indirect effect on WTC through the serial chain of AI self-efficacy and FLE.

Research on FLE has grown rapidly since Dewaele and MacIntyre (2014) proposed the construct and developed its measurement instrument. Cross-cultural validation studies have confirmed FLE's positive associations with effort, persistence, and language proficiency across diverse learner populations (Dewaele and Alfawzan, 2018; Dewaele et al., 2018). In Chinese EFL contexts specifically, FLE has been found to predict participation, oral performance, and overall academic achievement, even after controlling for the competing negative influence of FLA (Jiang and Dewaele, 2019; Li et al., 2020). The present model extends this line of research by proposing that AI tool use is an upstream antecedent of FLE, operating through the intermediate mechanism of self-efficacy as specified by CVT appraisal theory.

The CVT framework also illuminates why FLA may moderate rather than simply counterbalance FLE. According to Pekrun (2006), academic emotions interact dynamically: anxiety and enjoyment are not merely polar opposites but may coexist and influence each other's downstream effects. High anxiety can reduce the action tendency—the motivational-behavioral consequence—associated with enjoyment by competing for attentional resources and generating avoidance impulses. In the L2 domain, this suggests that even genuinely enjoyed AI practice may not translate into WTC if anxiety about face-to-face communication is sufficiently elevated. CVT therefore predicts not just that FLE promotes WTC, but that this promotion is contingent on the anxiety context in which communicative intentions are formed, providing theoretical grounding for the moderated mediation model tested here.

Emerging empirical evidence corroborates these theoretical observations regarding the joint effects of anxiety and positive affect in L2 contexts. Wang et al. (2025) found that anxiety, motivation, and engagement constituted a complex interactive system among Chinese as a second language (CSL) learners, with anxiety attenuating motivational benefits in ways consistent with CVT's action-tendency mechanism. In an EFL teaching context, Hoang (2025) demonstrated that cultural-linguistic congruence moderated teacher emotion dynamics during AI implementation, suggesting that contextual factors mediate the extent to which AI-related positive affect translates into communicative behavior. Abdelaty et al. (2025) further showed that learners' self-regulation capacity mediated the relationship between feedback conditions and L2 writing achievement, implying that anxiety and related affective variables operate through motivational-regulatory pathways that parallel the FLE-to-WTC mechanism modeled in the present study.

### 2.4 Foreign language anxiety as a moderator

Foreign language anxiety (FLA) is one of the most extensively documented negative emotions in L2 research. Horwitz et al. (1986) conceptualized FLCA as a distinct, situation-specific anxiety syndrome comprising communication apprehension, test anxiety, and fear of negative evaluation. FLA is particularly prevalent among Chinese EFL learners, shaped by high-stakes examination culture, Confucian face concerns, and the perceived social costs of public language errors (Horwitz, 2001, 2010).

Although FLE and FLA are empirically separable and often negatively correlated, they coexist within many learners, creating a so-called “bittersweet” experience of language learning (Dewaele and MacIntyre, 2014). Research has found that when anxiety is high, the positive motivational impact of enjoyment on communicative engagement is weakened: anxious learners may experience FLE during AI-assisted practice yet remain inhibited in face-to-face communication. This suppression logic is consistent with attentional control theory, which predicts that anxiety diverts cognitive resources away from goal-directed engagement (Khajavy et al., 2018; Li et al., 2020). Accordingly:

**H7:** FLA moderates the FLE-to-WTC relationship such that the positive association between FLE and WTC is weaker at higher levels of FLA.
**H8:** The serial indirect effect of AI tool use on WTC via AI self-efficacy and FLE is moderated by FLA (moderated serial mediation).

### 2.5 Willingness to communicate in Chinese EFL contexts

Willingness to communicate (WTC) emerged as a pivotal construct in second language acquisition research following MacIntyre et al.'s (1998) seminal pyramid model. Originally conceptualized in first language contexts as a relatively stable personality disposition, WTC was reconceptualised by MacIntyre and colleagues as a dynamic, situation-specific orientation reflecting the learner's readiness to engage in L2 communication when given the opportunity. The pyramid model situates WTC at the apex of a hierarchical system of antecedents, with immediate communication intentions directly influenced by situational self-confidence—comprising perceived communicative competence and L2 anxiety—and by the desire to affiliate with a particular interlocutor. Deeper in the pyramid lie layers of relatively enduring individual differences, including motivation, attitudes, and inter-group orientation. This layered architecture makes WTC simultaneously sensitive to in-the-moment psychological states and to chronic dispositional tendencies, giving the construct both theoretical richness and practical tractability.

Since the publication of MacIntyre et al.'s (1998) model, a substantial body of research has documented the predictors and consequences of WTC in EFL settings. Higher WTC is associated with greater communicative initiation, longer speaking turns, broader vocabulary use, and faster long-term proficiency development (Khajavy et al., 2018). Conversely, learners with low WTC tend to avoid communicative opportunities, reinforcing the anxiety and competence deficits that initially suppressed their willingness to speak. This self-perpetuating cycle is particularly well documented in East Asian EFL contexts, where instructional emphasis on grammatical accuracy, peer evaluation norms, and Confucian face concerns collectively discourage communicative risk-taking (Dewaele and MacIntyre, 2014; Jiang and Dewaele, 2019).

In the specific context of Chinese university EFL learners, WTC has been identified as a key bottleneck in English proficiency development. Despite years of formal instruction, many Chinese EFL students report reluctance to speak English in class or with native speakers even when their receptive skills are adequate. Qualitative and mixed-methods research has attributed this gap between receptive and productive competence partly to elevated FLA, partly to limited authentic communicative practice, and partly to curricular structures that prioritize exam preparation over communicative fluency (Horwitz, 2010; Li et al., 2020). The emergence of AI tools as always-available, low-stakes conversation partners therefore represents a potentially transformative development: for the first time, Chinese EFL learners can accumulate extended communicative practice experience without the evaluative threat of peer or teacher judgement, theoretically creating the conditions for both higher self-efficacy and greater WTC.

The relationship between emotional states and WTC has been extensively studied in recent years, catalyzed by the positive psychology turn in SLA research (Dewaele et al., 2019). Foreign language anxiety is the most widely researched negative predictor of WTC (Horwitz et al., 1986; Horwitz, 2001), with meta-analytic evidence confirming its robust negative association with communicative engagement across cultures and proficiency levels. More recently, positive emotions—particularly FLE—have been shown to exert independent positive effects on WTC over and above the negative influence of anxiety (Dewaele and MacIntyre, 2014; Khajavy et al., 2018). These complementary findings suggest that WTC is co-determined by both the presence of positive affect and the absence of anxiety inhibition, motivating the moderated mediation design of the present study, which explicitly models how AI-generated enjoyment may differentially promote WTC depending on the learner's anxiety baseline.

Critically, no prior study has examined AI tool use as an upstream antecedent of the affective processes that govern WTC. Existing research has mapped the immediate predictors of WTC (self-confidence, anxiety, enjoyment) and has examined technology-use correlates of language outcomes, but the mechanism linking AI tool use specifically to WTC through the dual intermediaries of self-efficacy and enjoyment remains untested. The present study fills this gap by positioning AI tool use at the beginning of the causal chain that ultimately determines whether Chinese EFL learners initiate English communication, grounding this chain theoretically in both SCT's competence-building account and CVT's emotion-generation account.

### 2.6 The present study

The foregoing review motivates a moderated serial mediation model in which AI tool use predicts WTC through two interconnected psychological mechanisms: (1) the accumulation of competence beliefs (AI self-efficacy, consistent with SCT), and (2) the generation of positive academic emotions (FLE, consistent with CVT). These two mechanisms are themselves sequentially related: learners who develop stronger AI self-efficacy appraise their AI-mediated learning activities as both controllable and valuable, leading to greater FLE. FLE, in turn, expands learners' behavioral engagement, increasing their WTC. AI self-efficacy also exerts a direct influence on WTC through the perceived competence pathway specified in MacIntyre et al.'s (1998) pyramid model. Foreign language anxiety is introduced as a moderator of the FLE-to-WTC link because, as CVT predicts, anxiety competes with enjoyment for attentional resources and generates avoidance impulses that weaken enjoyment's positive effect on communicative behavior.

The integrated model thus simultaneously encompasses an enabler pathway (AI use → self-efficacy → FLE → WTC) and a boundary condition (FLA attenuating the enjoyment-WTC transition). Two research questions guide the empirical investigation. RQ1 asks whether AI tool use predicts WTC through the proposed serial mediation chain via AI self-efficacy and FLE. RQ2 asks whether this chain is moderated by FLA such that the beneficial effect of enjoyment on WTC is weaker for learners experiencing high anxiety. Eight directional hypotheses are derived from these questions and tested using CBSEM in a sample of 420 Chinese EFL undergraduates drawn from two geographically distinct universities.

**Figure 1:**
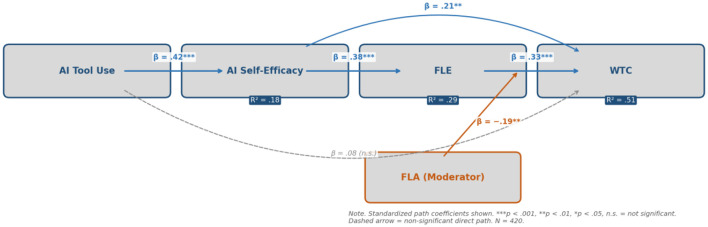
Structural equation model with standardized path coefficients. ***p < 0.001, **p < 0.01. Dashed arrow = non-significant path. *R*^2^ values in brackets. *N* = 420.

Figure 1 (see Section 4) presents the integrated moderated serial mediation model with all proposed pathways. The eight hypotheses together describe how AI tool use may both directly and indirectly cultivate WTC through a positive affective chain, and why this chain may be attenuated for learners experiencing high language anxiety.

## 3 Method

### 3.1 Participants and procedure

Participants were EFL undergraduates recruited from two Chinese universities: a comprehensive university in Zhejiang Province (eastern China) and a polytechnic university in Shaanxi Province (western China). The dual-site design was adopted to enhance geographic and socioeconomic diversity, given documented regional differences in English proficiency and technology access within mainland China. The study protocol was reviewed and approved by the Ethics Review Committee of the Education Division, Sichuan Academy of Social Sciences; data collection at the two participating universities in Zhejiang and Shaanxi Provinces was additionally approved by their respective institutional review boards prior to data collection. Participation was voluntary, and all respondents provided written informed consent.

A self-report online questionnaire was administered between March and May 2025 via the Wenjuanxing platform. Research assistants distributed survey links during regular English classes with permission from course instructors. Respondents were assured of anonymity and could withdraw at any point. A total of 480 questionnaires were submitted. The Wenjuanxing platform records a response only upon final submission; survey links that were opened but not submitted are not stored, and the option to save partial responses was not enabled. All core measurement items were set as mandatory, so every submitted questionnaire contained complete data on the study variables. Prior to analysis, all submissions were screened for careless responding: 21 questionnaires exhibiting straight-lining (i.e., selecting the same response option across all items) and 39 that failed at least one of two embedded attention-check items were excluded, yielding a final analytical sample of 420 participants (Eastern site: *n* = 214; Western site: *n* = 206). The sample included 268 females (63.8%) and 152 males (36.2%), with a mean age of 20.4 years (SD = 1.32). Self-rated English proficiency was predominantly intermediate (41.7%) and upper-intermediate (35.2%). Self-rated English proficiency was reported on a four-level scale (beginner, intermediate, upper-intermediate, advanced) corresponding to learners' most recent institutional English-course placement and their self-assessment against CEFR-aligned “can-do” descriptors. Self-report was adopted because standardized proficiency scores were not uniformly available across the two institutions; although self-ratings correlate only imperfectly with objective measures, they provide an acceptable proficiency index for sample-description purposes in large-scale survey research. Proficiency and year of study were recorded for sample description only and were not entered as analytic variables; they are retained in Table 1 for transparency.

**Table 1:** Sample demographic characteristics.

Characteristic	*n*	%
Gender		
Female	268	63.8
Male	152	36.2
Year of study		
Year 1	93	22.1
Year 2	120	28.6
Year 3	130	31.0
Year 4+	77	18.3
English proficiency (self-rated)		
Beginner	22	5.2
Intermediate	175	41.7
Upper-intermediate	148	35.2
Advanced	75	17.9
University region		
Eastern China	214	51.0
Western China	206	49.0

Total N = 420.

The choice of Chinese universities as the research site was deliberate. China has invested heavily in technology-enhanced English education, with AI tools increasingly endorsed in national curriculum guidelines and integrated into major domestic online learning platforms. The Zhejiang–Shaanxi dual-site design was adopted to capture meaningful variation in regional development level, internet penetration, and exposure to authentic English input outside the classroom, thereby providing ecological diversity that single-site studies lack. Pre-liminary analyses confirmed that participants at the two sites did not differ significantly on any focal construct (all ps > 0.10), justifying pooled analysis while retaining the dual-site design as a guard against site-specific confounds.

Prior to main data collection, the questionnaire was piloted with 42 undergraduates from a third university not included in the main study to check item clarity, estimated completion time, and comprehension of AI-tool-related vocabulary. Minor wording revisions were made to three items following pilot feedback; specifically, illustrative examples of AI tools were updated to include locally popular applications (e.g., Kimi, Doubao) alongside internationally familiar tools such as ChatGPT and DeepL. The revised instrument was reviewed by a panel of three bilingual EFL researchers before final deployment, and cross-cultural equivalence between English-origin constructs (FLE, FLA, WTC) and their Chinese adaptations was verified through confirmatory factor analysis in the main data prior to structural model estimation.

### 3.2 Measures

All items were translated into Chinese using the standard back-translation procedure (Brislin, 1970). A bilingual expert panel verified conceptual equivalence prior to administration. Items used five-point Likert scales (1 = strongly disagree, 5 = strongly agree) unless otherwise stated.

#### 3.2.1 AI tool use

Frequency and variety of AI tool use for English learning were assessed with eight items adapted from Çobanogullari and Özbek (2025). Sample item: “I regularly use AI tools (e.g., ChatGPT, DeepL) to practice English.” Cronbach's α = 0.83; composite reliability (CR) = 0.90. A second representative item is: “I use AI tools to check or improve my English writing.” Although ChatGPT is not officially available in mainland China, university students routinely access it on their own through third-party platforms, and DeepL is directly accessible; the examples therefore reflect tools genuinely used by the target population. No tool access was provided or mandated by the research team, and all usage reflected participants' self-initiated choices.

#### 3.2.2 AI self-efficacy

Ten items assessed learners' confidence in using English with AI tool support, adapted from Graham (2022) and Raoofi et al. (2012) with items reworded to reference AI-mediated contexts. Sample item: “When I use AI tools for English practice, I feel confident in my ability to communicate in English.” α = 0.88; CR = 0.94. Additional sample item: “I am confident that I can use AI tools to prepare myself for speaking in English.”

#### 3.2.3 Foreign language enjoyment (FLE)

The 21-item FLE scale developed by Dewaele and MacIntyre (2014) was used, measuring positive emotions experienced in the EFL classroom and during self-directed English study. Sample item: “I enjoy using English in class.” α = 0.86; CR = 0.97. Sample item: “I enjoy my English learning and often find it fun.”

#### 3.2.4 Foreign language anxiety (FLA)

Fourteen items from the shortened version of the Foreign Language Classroom Anxiety Scale (FLCAS; Horwitz et al., 1986) were used. Items were adapted to encompass both classroom and AI-tool-related contexts. Sample item: “I feel nervous when I am speaking English, even when using an AI tool.” α = 0.81; CR = 0.94. Additional sample item: “I worry about making mistakes when I have to communicate in English.”

#### 3.2.5 Willingness to communicate (WTC)

WTC was assessed with eleven items adapted from MacIntyre et al. (1998) and validated for the Chinese EFL context by Khajavy et al. (2018). Sample item: “I am willing to start a conversation in English with a classmate.” α = 0.89; CR = 0.96. Additional sample item: “I am willing to give a short talk in English in front of the class.” The complete item pool for all five scales is provided as Supplementary material.

### 3.3 Analytic strategy

Data were analyzed using covariance-based structural equation modeling (CBSEM) with maximum likelihood robust (MLR) estimation in Mplus 8.8 (Muthén and Muthén, 1998–2017). The analytic sequence followed established SEM conventions (Hair et al., 2019): (1) confirmatory factor analysis (CFA) to evaluate the measurement model; (2) assessment of convergent validity (factor loadings > 0.60; AVE > 0.50; CR > 0.70) and discriminant validity using Heterotrait-Monotrait (HTMT) ratios below 0.85; (3) structural path estimation with simultaneous testing of direct and indirect effects; (4) bootstrapped mediation analysis (10,000 replications) to obtain bias-corrected 95% confidence intervals for indirect effects (Hu and Bentler, 1999); and (5) product-of-coefficients test for moderated mediation (FLA as moderator). Missing data were minimal (1.8% of data points), confined to optional demographic items used for sample description only; all core measurement items were set as mandatory and were therefore complete. Full information maximum likelihood (FIML) was retained as the estimator for the structural models. Common method bias was evaluated by comparing the hypothesized five-factor model against a single-factor model. Both the measurement and structural models were over-identified (measurement model df = 938; structural model df = 942), with the number of freely estimated parameters well below the number of non-redundant elements in the input covariance matrix; model identification was therefore not a concern.

## 4 Results

### 4.1 Participant characteristics

A total of 480 questionnaires were submitted across two sampling sites—one eastern university (Zhejiang Province) and one western university (Shaanxi Province). After excluding 21 responses for straight-lining (careless responding) and 39 for failing attention-check items, the final sample comprised 420 valid responses (Eastern: *n* = 214; Western: *n* = 206). Participants included 268 females (63.8%) and 152 males (36.2%), with a mean age of 20.4 years (SD = 1.32). Self-rated English proficiency was predominantly intermediate (41.7%) and upper-intermediate (35.2%). Regarding AI tool use frequency, the majority reported weekly (34.8%) or daily use (28.6%), with ChatGPT-type tools (68.3%) and translation tools such as DeepL (57.6%) being the most common.

### 4.2 Descriptive statistics and bivariate correlations

Table 2 presents means, standard deviations, reliability coefficients, and zero-order correlations for all study variables. All scales demonstrated adequate internal consistency (α range: 0.81–0.89). Bivariate correlations were consistent with hypothesized directions: AI tool use was positively associated with AI Self-Efficacy (*r* = 0.48, *p* < 0.001), FLE (*r* = 0.41, p < 0.001), and WTC (*r* = 0.39, *p* < 0.001). Foreign language anxiety was negatively correlated with FLE (*r* = −0.37, *p* < 0.001) and WTC (*r* = −0.44, *p* < 0.001). Average variance extracted (AVE) exceeded 0.50 for all constructs (range: 0.52–0.68), and Heterotrait–Monotrait (HTMT) ratios were all below 0.85, supporting discriminant validity. The square root of the AVE for each construct (reported in parentheses on the diagonal of Table 2: 0.73, 0.79, 0.76, 0.72, and 0.82, respectively) exceeded that construct's correlations with all other constructs, satisfying the Fornell–Larcker criterion for discriminant validity.

**Table 2:** Descriptive statistics, reliabilities, and zero-order correlations.

Variable	*M*	SD	α	CR	AVE	1	2	3	4	5
1. AI tool use	3.52	0.71	0.83	0.90	0.54	(0.73)				
2. AI self-efficacy	3.67	0.68	0.88	0.94	0.62	0.48^***^	(0.79)			
3. FLE	3.74	0.74	0.86	0.97	0.58	0.41^***^	0.52^***^	(0.76)		
4. FLA	3.04	0.82	0.81	0.94	0.52	−0.22^***^	−0.31^***^	−0.37^***^	(0.72)	
5. WTC	3.87	0.76	0.89	0.96	0.68	0.39^***^	0.46^***^	0.51^***^	−0.44^***^	(0.82)

N = 420. M, mean; SD, standard deviation; α, Cronbach's alpha; CR, composite reliability; AVE, average variance extracted. FLA scores are reverse-coded such that higher scores indicate higher anxiety. ^***^p < 0.001. Values in parentheses on the diagonal are the square root of the AVE.

### 4.3 Confirmatory factor analysis

A five-factor CFA was conducted to assess the measurement model. The hypothesized structure fit the data well: $\chi_{\left(938\right)}^{2}$ = 1,714.3, χ^2^/df = 1.83, RMSEA = 0.044, 90% CI [0.040, 0.048], CFI = 0.967, TLI = 0.963, SRMR = 0.052. All standardized factor loadings exceeded 0.60 (range: 0.62–0.86, all ps < 0.001). The five-factor model fit significantly better than a single-factor alternative [$\Delta \chi_{\left(10\right)}^{2}$ = 3,847.5, *p* < 0.001], indicating that common method bias was not a serious concern (see Table 3). As a complementary check, a Harman's single-factor test was conducted: the first unrotated factor accounted for 31.6% of the total variance, well below the 50% threshold, reinforcing the nested-model comparison in indicating that common method bias was unlikely to have distorted the findings.

**Table 3:** Confirmatory factor analysis model fit indices.

Index	Value	Criterion	Met
χ^2^/df	1.83	< 3.0	Yes
RMSEA	0.044	< 0.060	Yes
90% CI [RMSEA]	[0.040, 0.048]	Upper < 0.08	Yes
CFI	0.967	>0.950	Yes
TLI	0.963	>0.950	Yes
SRMR	0.052	< 0.080	Yes

N = 420. Estimation: MLR in Mplus 8.8.

### 4.4 Structural model and direct effects (H1–H4)

The structural model fit was acceptable: $\chi_{\left(942\right)}^{2}$ = 1,728.6, χ^2^/df = 1.84, RMSEA = 0.045, CFI = 0.966, TLI = 0.962, SRMR = 0.055. Figure 1 presents the standardized path coefficients. Full path statistics are reported in Table 4. H1 was supported: AI tool use significantly predicted AI Self-Efficacy (β = 0.42, SE = 0.05, *p* < 0.001). H2 was supported: AI Self-Efficacy significantly predicted FLE (β = 0.38, SE = 0.06, *p* < 0.001). H3 was supported: FLE significantly predicted WTC (β = 0.33, SE = 0.05, *p* < 0.001). H4 was supported: AI Self-Efficacy exerted a significant direct effect on WTC (β = 0.21, SE = 0.06, *p* = 0.001). The direct effect of AI Tool Use on WTC was non-significant after controlling for mediators (β = 0.08, SE = 0.06, *p* = 0.192), suggesting full mediation. The model explained 18% of the variance in AI Self-Efficacy, 29% in FLE, and 51% in WTC.

**Table 4:** Structural path coefficients.

Path	β	SE	*p*	Hypothesis	Decision
AI Tool Use → AI self-efficacy	0.42	0.05	< 0.001	H1	Supported
AI self-efficacy → FLE	0.38	0.06	< 0.001	H2	Supported
FLE → WTC	0.33	0.05	< 0.001	H3	Supported
AI self-efficacy → WTC (direct)	0.21	0.06	0.001	H4	Supported
AI tool use → WTC (direct, controlled)	0.08	0.06	0.192	—	n.s.

Standardized β coefficients. SE, standard error; n.s., not significant. N = 420.

### 4.5 Mediation effects (H5–H6)

**Figure 2:**
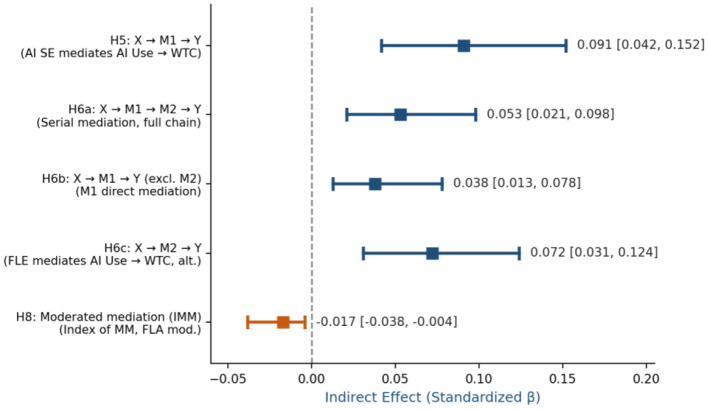
Forest plot of bootstrapped indirect and moderated mediation effects with 95% confidence intervals (10,000 replications). IMM, index of moderated mediation. Bars not crossing zero indicate significant effects. *N* = 420.

Bootstrapped mediation analyses (10,000 replications) were used to test the indirect effects. H5 was supported: the indirect effect of AI Tool Use on WTC via AI Self-Efficacy was significant [β = 0.091, SE = 0.027, 95% CI (0.042, 0.152)]. H6 was supported: the serial indirect effect through the chain AI Tool Use → AI Self-Efficacy → FLE → WTC was also significant [β = 0.053, SE = 0.019, 95% CI (0.021, 0.098)]. All 95% CIs excluded zero (see Table 5 and Figure 2).

**Table 5:** Bootstrapped indirect effects.

Indirect path	β	SE	95% CI LL	95% CI UL	H
AI Use → AI self-efficacy → WTC	0.091	0.027	0.042	0.152	H5 ✓
AI Use → AI self-efficacy → FLE → WTC (serial)	0.053	0.019	0.021	0.098	H6 ✓
AI Use → FLE → WTC (single mediator)	0.072	0.023	0.031	0.124	—
AI self-efficacy → FLE → WTC	0.038	0.016	0.013	0.078	—

Bootstrap replications = 10,000. LL, lower limit; UL, upper limit. CI not containing zero = statistically significant indirect effect. N = 420.

### 4.6 Moderated mediation: role of foreign language anxiety (H7–H8)

**Figure 3:**
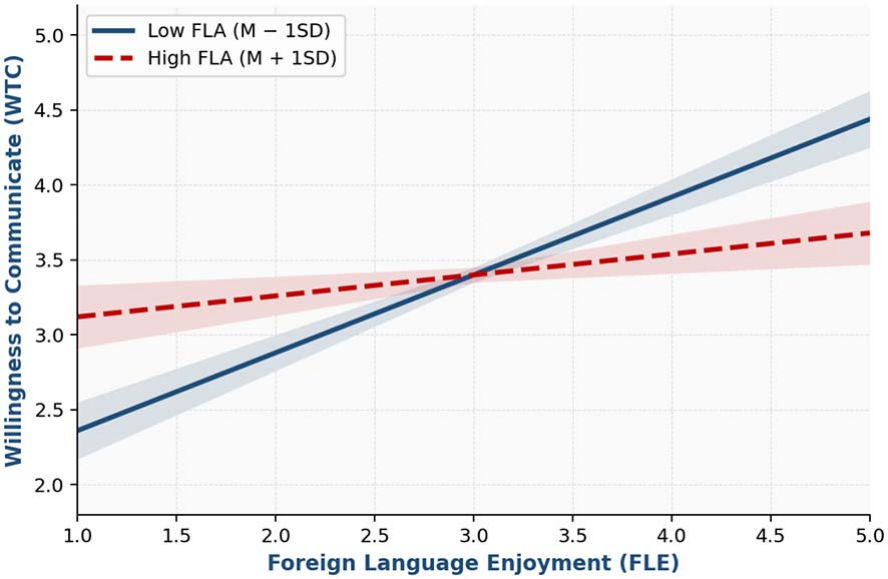
Simple slopes of the effect of foreign language enjoyment (FLE) on willingness to communicate (WTC) at low (*M* – 1SD) and high (*M* + 1SD) levels of foreign language anxiety (FLA). Shaded bands = ±1 SE. *N* = 420.

To test H7, the mean-centered interaction term FLE × FLA was entered into the structural model. The interaction was significant (β = −0.19, SE = 0.06, *p* = 0.002), supporting H7: the positive effect of FLE on WTC was weaker at higher levels of FLA. Simple slopes analysis revealed that the FLE → WTC slope was significant at low FLA (*M* – 1SD: β = 0.52, SE = 0.07, *p* < 0.001) but attenuated and non-significant at high FLA (*M* + 1SD: β = 0.14, SE = 0.08, *p* = 0.082), as illustrated in Figure 3. Full moderation and moderated-mediation statistics appear in Table 6. H8 was also supported: the index of moderated mediation (IMM) was significant [IMM = −0.017, SE = 0.009, 95% CI (−0.038, −0.004)], confirming that FLA moderated the serial indirect effect. For completeness, the conditional effects with confidence intervals were: low FLA (*M* – 1SD), β = 0.52, 95% CI [0.38, 0.66]; high FLA (*M* + 1SD), β = 0.14, 95% CI [−0.02, 0.30].

**Table 6:** Moderation and moderated mediation results.

Effect	β/IMM	SE	95% CI LL	95% CI UL	H
FLE × FLA interaction	−0.19	0.06	−0.31	−0.07	H7 ✓
Simple slope: low FLA (*M* – 1SD)	0.52	0.07	0.38	0.66	*p* < 0.001
Simple slope: high FLA (*M* + 1SD)	0.14	0.08	−0.02	0.30	*p* = 0.082
IMM (moderated mediation)	−0.017	0.009	−0.038	−0.004	H8 ✓

Variables mean-centered prior to computing the interaction. IMM, index of moderated mediation. 95% CIs via 10,000 bootstrap replications. N = 420.

### 4.7 Summary of hypothesis test results

Table 7 summarizes the results of hypothesis testing across all eight hypotheses.

**Table 7:** Summary of hypothesis test results.

H	Statement	Outcome
H1	AI tool use → AI self-efficacy (+)	Supported, β = 0.42^***^
H2	AI self-efficacy → FLE (+)	Supported, β = 0.38^***^
H3	FLE → WTC (+)	Supported, β = 0.33^***^
H4	AI self-efficacy → WTC (+, direct)	Supported, β = 0.21^**^
H5	AI tool use → AI Self-Efficacy → WTC	Supported, 95% CI [0.042, 0.152]
H6	AI tool use → AI self-efficacy → FLE → WTC (serial)	Supported, 95% CI [0.021, 0.098]
H7	FLA moderates FLE → WTC (negative moderation)	Supported, β = −0.19^**^
H8	FLA moderates serial indirect effect (IMM ≠ 0)	Supported, IMM = −0.017, 95% CI [−0.038, −0.004]

^***^p < 0.001, ^**^p < 0.01. Bootstrap CIs based on 10,000 replications. N = 420.

## 5 Discussion

The present study tested a moderated serial mediation model in which AI tool use predicted willingness to communicate sequentially through AI self-efficacy and foreign language enjoyment, with foreign language anxiety moderating the terminal link. Drawing on social cognitive theory and control-value theory, and using CBSEM with a geographically diverse Chinese EFL sample (N = 420), all eight hypotheses were supported. This section discusses each set of findings, their theoretical contributions, and practical implications.

### 5.1 Direct and indirect effects of AI tool use on communicative outcomes

The finding that AI tool use predicts AI self-efficacy (H1: β = 0.42) accords with Bandura's (1997) argument that structured mastery experiences are the most potent source of self-efficacy beliefs. AI tools—which provide immediate, calibrated, and emotionally safe feedback—may offer a uniquely rich mastery environment: learners can attempt communicative tasks repeatedly without the social risk that characterizes live classroom interaction. This finding extends Graham's (2022) review of self-efficacy processes in language learning by demonstrating that AI-mediated practice contexts are effective platforms for self-efficacy cultivation, consistent with earlier work on CALL and self-efficacy (Raoofi et al., 2012).

The direct effect of AI self-efficacy on WTC (H4: β = 0.21) replicates and extends MacIntyre et al.'s (1998) model, which positioned perceived competence as a proximal antecedent of WTC. In the present study, this relationship holds even after controlling for the indirect pathway through FLE, suggesting that self-efficacy has both direct and enjoyment-mediated routes to WTC. The full mediation of AI Tool Use → WTC (direct path non-significant at β = 0.08, p = 0.192) indicates that the technology-to-communication link is primarily psychological rather than direct—AI tools matter because they change how learners think and feel about English, not through some unmediated stimulus-response mechanism.

The magnitude of the H1 path coefficient (β = 0.42) is noteworthy in comparative perspective. Prior studies of non-AI CALL technologies—including multimedia language learning software, online writing platforms, and synchronous computer-mediated communication—have typically reported self-efficacy effects in the small-to-medium range (Raoofi et al., 2012). The stronger association observed here may reflect AI tools' unique capacity for individualized, adaptive feedback that exceeds what conventional CALL technologies can provide. Whereas earlier tools delivered scripted feedback based on pre-programmed error categories, contemporary AI language tools generate contextually sensitive, linguistically sophisticated responses that more closely simulate the formative guidance of a human tutor. This qualitative difference in feedback richness may explain the stronger self-efficacy return on AI practice investment relative to earlier technology generations.

It is also worth considering the theoretical implications of the non-significant direct path from AI Tool Use to WTC (β = 0.08, *p* = 0.192). This full mediation pattern suggests that AI tools *per se* do not directly motivate communication; rather, their communicative benefits are fully channeled through the psychological mechanisms of self-efficacy and enjoyment. This has an important practical corollary: simply providing learners with access to AI tools is unlikely to produce WTC gains in the absence of conditions that support competence belief formation and emotional engagement. Instructional designs that merely expose students to AI resources without scaffolding self-efficacy development—for example, without goal-setting activities, reflection prompts, or progress feedback—may fail to realize the communicative potential that the technology affords. The mediation findings therefore underscore the need for pedagogically intentional AI integration rather than passive technology provision. This full-mediation pattern is itself theoretically informative. That AI tool use exerts no detectable direct effect on WTC once the affective–cognitive chain is accounted for indicates that AI tools do not raise communicative readiness through mere exposure or convenience; their influence is realized entirely through the psychological resources they help build—competence beliefs and enjoyment. Practically, this implies that deploying AI tools without attending to learners' efficacy and emotional experience is unlikely to move WTC, a point developed in the practical implications below.

### 5.2 The serial mediation chain through enjoyment

The confirmed serial indirect effect [H6: β = 0.053, 95% CI (0.021, 0.098)] represents the study's most theoretically novel contribution. Prior work established that FLE predicts WTC (Dewaele and MacIntyre, 2014; Jiang and Dewaele, 2019) and that self-efficacy predicts WTC (Khajavy et al., 2018), but the sequential self-efficacy → enjoyment pathway had not been explicitly tested in an AI learning context. In the present model, AI self-efficacy predicts FLE (H2: β = 0.38), which in turn predicts WTC (H3: β = 0.33). This ordering is consistent with CVT appraisal logic (Pekrun, 2006): when learners believe they are capable of succeeding at AI-mediated language tasks (control appraisal), they appraise those tasks as intrinsically pleasurable (enjoyment), and this positive affect subsequently broadens their readiness to communicate (Pekrun and Perry, 2014). These findings also speak to the emerging literature on AI-mediated communication. Recent work tracking EFL learners' interactions with generative AI reports that willingness to communicate with AI is itself dynamic and tied to learners' acceptance of, and affective responses to, the tool (Peng and Liang, 2025); the present results extend this line by showing that AI tool use is associated not only with communication directed at AI but, indirectly, with willingness to communicate in authentic L2 settings, via self-efficacy and enjoyment.

Taken together, the mediation results—the simple indirect path via AI self-efficacy (H5) and the serial chain via AI self-efficacy and foreign language enjoyment (H6)—position AI tools as emotion-shaping affordances in EFL learning. The technology does not produce WTC directly; rather, it initiates a positive psychological cascade—competence beliefs fuel enjoyment, enjoyment fuels communication—that collectively accounts for a substantial share of WTC variance (51%). This cascade metaphor invites future researchers to examine whether similar chains operate in other technology-enhanced learning contexts, and whether the self-efficacy → enjoyment → WTC sequence is specific to the AI context or reflects a more general positive psychology trajectory in L2 learning (cf. Dewaele et al., 2019; Li et al., 2020).

The parallel between the present findings and positive psychology research in SLA is worth noting explicitly. The flourishing of positive psychology applications in L2 learning—documented by Dewaele et al. (2019)—has consistently emphasized that positive emotions are not merely pleasant by-products of successful language use but active causal contributors to further engagement and achievement. The present serial mediation chain, in which self-efficacy generates enjoyment and enjoyment generates WTC, is structurally consistent with the broaden-and-build logic from affective science: positive emotions broaden cognitive and behavioral repertoires, building enduring psychological resources—including heightened WTC—that outlast any individual affective episode (Pekrun and Perry, 2014). Applied to the AI context, this suggests that each productive, enjoyable AI practice session may incrementally strengthen learners' communicative dispositions, ultimately lowering the threshold for spontaneous L2 communication in non-AI contexts. Future diary or ecological momentary assessment studies are well positioned to test whether AI-generated enjoyment episodes accumulate into dispositional WTC growth over time.

### 5.3 Moderation by foreign language anxiety

The moderation findings reveal an important caveat to the otherwise uniformly positive picture described above. For learners high in FLA, the FLE → WTC link was substantially attenuated [simple slope at high FLA: β = 0.14, ns, 95% CI (−0.02, 0.30)], supporting H7, relative to learners low in FLA (β = 0.52, *p* < 0.001). This pattern is consistent with attentional control theory and with Horwitz's (2001) contention that anxiety can override other psychological resources, disrupting the translation of positive affect into communicative behavior. Notably, the significant index of moderated mediation [IMM = −0.017, 95% CI (−0.038, −0.004)], supporting H8, confirms that anxiety moderates not only the direct FLE-to-WTC step but the entire serial indirect pathway: the beneficial cascade triggered by AI tool use is partially blocked for anxious learners. Attentional control theory (Eysenck et al., 2007) offers a precise account of this attenuation: anxiety diverts limited attentional and working-memory resources toward threat monitoring and self-relevant worry, reducing the cognitive capacity available for translating a positive affective state into the goal-directed act of initiating communication. For highly anxious learners, enjoyment may therefore be experienced without being converted into the approach behavior that WTC represents, because the resources required to act on that enjoyment are consumed by anxiety-related processing.

This finding bridges the positive psychology stream in SLA—which has emphasized FLE and other positive emotions (Dewaele and Alfawzan, 2018; Dewaele and MacIntyre, 2014)—and the longer-standing anxiety tradition (Horwitz et al., 1986; Horwitz, 2010). Rather than treating FLE and FLA as simple opposites, the present model demonstrates that they interact: enjoyment cannot fully compensate for the inhibitory effect of anxiety on WTC. This “bittersweet” interference (Dewaele and MacIntyre, 2014) appears particularly pronounced in the Chinese EFL context, where face concerns and performance pressure sustain high baseline anxiety levels even as AI tools generate genuine positive affect during practice.

The clinical and educational implications of the moderation finding merit closer consideration. The data indicate that highly anxious learners—who arguably stand to benefit most from the positive affective cascade initiated by AI tools—are precisely those for whom the cascade is most disrupted at the FLE-to-WTC transition. This creates a paradox of potential: AI tools generate genuine enjoyment even in anxious learners (the FLE–FLA correlation was negative but moderate, *r* = −0.37), yet this enjoyment fails to translate into communicative willingness when anxiety is high. Pedagogically, this suggests that AI tool integration should be complemented with evidence-based anxiety interventions—such as cognitive reappraisal training, autonomy-supportive teaching, and structured communicative risk-taking activities—that directly target the anxiety-WTC bottleneck. The goal would be to remove the anxiety-related blockage at the FLE-WTC transition point, allowing the AI-generated positive affect to complete its full trajectory toward communicative engagement.

### 5.4 Theoretical contributions

This study makes three theoretical contributions. First, it integrates social cognitive theory and control-value theory within a unified empirical model, demonstrating that SCT's competence-building mechanisms and CVT's appraisal-based emotion generation are not merely compatible but sequentially linked: self-efficacy (SCT) precedes and predicts enjoyment (CVT), which then predicts communication. This theoretical integration is underspecified in prior work and suggests a productive direction for multi-theory SLA models. Second, by placing AI tool use as the exogenous predictor, the study positions AI as an antecedent of SCT and CVT processes rather than a mere correlate of outcomes, advancing theoretical understanding of how educational technology shapes the psychological conditions for L2 communication. Third, the moderated serial mediation design reveals a previously undocumented boundary condition: the serial positive cascade is most effective precisely for learners who least require it (low-anxiety), while remaining partially blocked for those who would benefit most (high-anxiety). This paradox calls for targeted theoretical elaboration of how affective enablers and inhibitors interact across different learning environments.

A fourth, methodological contribution deserves acknowledgment. The use of CBSEM with MLR estimation—rather than the partial least squares SEM approach increasingly common in technology acceptance research—provides a rigorous test of the mediation architecture by simultaneously estimating measurement and structural parameters, accounting for measurement error, and enabling stringent model fit evaluation. The excellent fit indices (CFI = 0.967, RMSEA = 0.044) provide strong evidence that the proposed five-factor structure adequately represents the covariation among constructs. The detection of moderated mediation at the full SEM level—using the product-of-coefficients approach with latent variables—also addresses a methodological gap in prior WTC research, which has predominantly relied on regression-based path models that do not account for measurement error in latent constructs. Future research should consider latent moderated structural equation approaches that estimate interaction terms without product indicators, providing an even more rigorous evaluation of the moderated mediation architecture.

### 5.5 Practical implications

The findings carry several implications for EFL instructors and curriculum designers. First, integrating AI tools into regular language practice is likely to strengthen learners' self-efficacy and enjoyment, and thereby their communicative engagement. Instructors should therefore consider structuring AI tool use as a low-stakes rehearsal scaffold before live communicative activities—a “warm-up” that builds competence beliefs prior to higher-stakes interaction. Second, the finding that the AI-enjoyment-WTC cascade is attenuated for anxious learners suggests that AI tools alone are insufficient for highly anxious students. Emotionally responsive pedagogical strategies—including explicit anxiety-reduction instruction, collaborative AI use to reduce social isolation, and psychoeducation about language anxiety—should complement AI tool integration for this subgroup. Third, the regional similarity between eastern and western Chinese universities in the pattern of results (similar sample proportions and consistent internal consistency across sites) suggests that the model is reasonably generalizable within mainland Chinese tertiary EFL contexts, supporting policy-level recommendations for AI integration in Chinese university English curricula.

Three concrete instructional strategies follow from the moderation finding for high-anxiety learners. First, instructors can pair AI practice with low-stakes output tasks—for example, recorded mini-speeches or anonymous voice submissions—that allow anxious learners to convert AI-built confidence into communicative action without the evaluative pressure of live interaction. Second, brief mindfulness or cognitive-reappraisal training can help highly anxious students reinterpret communicative arousal as facilitative rather than threatening, freeing the attentional resources that anxiety would otherwise consume. Third, a scaffolding-fading program can be implemented for high-anxiety learners, progressing from fully AI-supported practice, through AI-assisted but instructor-monitored speech production, to unsupported authentic communication, so that the self-efficacy and enjoyment cultivated in the AI context are systematically transferred to live L2 settings.

Beyond classroom practice, the findings have implications for AI tool design at the institutional level. Universities adopting AI language learning platforms should consider incorporating features that explicitly support self-efficacy development—such as progress tracking dashboards, mastery certificates, and personalized skill gap feedback—alongside the conversational or generative AI features that are currently most visible. Equally, platforms should consider anxiety-sensitive onboarding protocols that normalize error and model strategic recovery from communication breakdowns, reducing the evaluative threat that sustains FLA. The moderated mediation finding also suggests that AI tools may be most beneficial when deployed in a scaffolded sequence that progresses from low-anxiety private AI interaction to AI-supported but instructor-monitored speech production, and finally to autonomous live communication. Such a sequence operationalizes the theoretical logic of the present model: build self-efficacy and enjoyment in the psychologically safe AI context first, then systematically transfer these resources to the live communicative contexts where WTC ultimately manifests.

The present findings also carry implications for language teacher professional development. As AI tools become integral to EFL classrooms, teachers must navigate complex pedagogical decisions about when and how to deploy AI scaffolding without undermining authentic communicative development (Zhang and Wang, 2025). Research on triadic professional support frameworks suggests that combining peer collaboration, AI-assisted reflection, and faculty mentorship cultivates more adaptive teaching identities—a model particularly relevant for EFL instructors balancing AI affordances with communicative pedagogy (Erbay-Çetinkaya, 2025). At the institutional level, cultivating epistemic trust in AI among educators and learners represents an important precondition: without confidence in AI systems' reliability and pedagogical alignment, both teachers and students may resist or superficially comply with AI-enhanced learning activities (Pandey et al., 2026). Scaling up AI tool use in tertiary EFL settings therefore requires sustained investment in professional development that addresses the affective and epistemic dimensions of AI adoption, not only technical deployment (Alreiahi and Alrwaished, 2025).

### 5.6 Limitations and future directions

Several limitations warrant acknowledgment. First, the cross-sectional design precludes causal inference. Although the hypothesized mediation sequence is grounded in theory, longitudinal or experimental designs—for example, randomly assigning students to AI-tool-augmented vs. conventional practice conditions—are needed to establish temporal precedence and directionality. Future studies could employ random experience sampling (ecological momentary assessment) to capture real-time fluctuations in self-efficacy, enjoyment, and WTC across AI practice sessions.

Second, the sample was restricted to undergraduate EFL learners at two Chinese universities. While the dual-site design provides initial evidence of geographic generalizability within China, the findings may not transfer to learners in other cultural or sociolinguistic contexts where face-saving norms, technology access, or educational systems differ. Replication with secondary school students, postgraduate learners, or learners in Southeast Asian and Middle Eastern EFL settings would strengthen external validity.

Third, all constructs were measured via self-report, introducing potential common method variance, though the CFA results suggest this was not a serious confound. Future research should supplement self-report with behavioral measures of WTC (e.g., speaking time in structured tasks) and objective indicators of AI tool use (e.g., platform-logged interaction frequency). Such multi-method designs would reduce reliance on retrospective judgements and improve the ecological validity of the constructs.

Fourth, the study treated AI tool use as a unidimensional frequency variable. Future research should disaggregate tool types—conversational chatbots, grammar checkers, pronunciation coaches—to examine whether different affordances activate the self-efficacy and enjoyment pathways differentially. It is plausible that chatbot-style tools that simulate communicative interaction generate stronger self-efficacy and enjoyment effects than passive tools such as AI-based dictionaries. A further measurement consideration concerns the alignment between constructs and contexts: AI self-efficacy was anchored to AI-mediated tasks, whereas the enjoyment measure indexes classroom-based foreign language enjoyment. Future research could employ enjoyment measures tailored to AI-mediated language learning to tighten this correspondence.

Finally, the present model focused on AI tool use, self-efficacy, and enjoyment as the key constructs linking technology to WTC. Other theoretically relevant variables—including growth mindset, L2 motivational self system, peer support, and instructor technophobia—were not examined and may moderate or mediate the pathways identified here. Richer future models that incorporate multiple antecedents and boundary conditions alongside AI tool use would provide a more complete picture of how the increasingly AI-saturated EFL learning environment shapes the affective and motivational foundations of communicative engagement. Such models might also attend to the possible curvilinear effects of AI tool use: excessive reliance on AI scaffolding could, over time, undermine autonomous communicative self-efficacy if learners come to attribute their language success to the tool rather than to their own growing competence.

## 6 Conclusion

The present study investigated how AI tool use shapes Chinese EFL learners' willingness to communicate through a positive affective chain. Grounded in social cognitive theory and control-value theory, a moderated serial mediation model was proposed and tested with covariance-based SEM using data from 420 undergraduates across eastern and western Chinese universities. The results confirmed that AI tool use predicts WTC through two complementary indirect paths: (1) via AI self-efficacy directly, and (2) via the serial chain of AI self-efficacy and foreign language enjoyment. Importantly, foreign language anxiety moderated the enjoyment-to-WTC link, attenuating the beneficial effect of enjoyment for highly anxious learners, and the full serial indirect effect was correspondingly weaker for this subgroup.

These findings enrich existing models of WTC by demonstrating that AI tools are not merely pedagogical conveniences but psychological affordances that initiate a cascade of competence beliefs and positive affect conducive to L2 communication. For educational practice, the results underscore the importance of positioning AI tool use as an enjoyment-enhancing scaffold within a broader emotionally responsive approach—one that simultaneously addresses the anxiety that continues to constrain communicative engagement for many Chinese EFL learners. Future longitudinal and experimental work should examine whether the self-efficacy and enjoyment mechanisms identified here operate causally, and whether targeted AI-integrated interventions can shift learners from the high-anxiety, low-WTC trajectory toward a more communicatively engaged experience of English learning.

## Data Availability

The raw data supporting the conclusions of this article will be made available by the authors, without undue reservation.
