# Supplementary material for: AI tool use, self-efficacy, foreign language enjoyment, and willingness to communicate: a moderated serial mediation model among Chinese EFL learners
Source: Front Psychol. 2026 Jul 21;17:1829287. doi: 10.3389/fpsyg.2026.1829287 (PMC13433450; doi:10.3389/fpsyg.2026.1829287)
Supplement: Supplementary file 1 [file Supplementary_file_1.docx]

**Supplementary Material S1**

**Measurement Instrument (Full Item Pool)**

*AI Tool Use, Self-Efficacy, Foreign Language Enjoyment, and Willingness to Communicate: A Moderated Serial Mediation Model*

**Instructions / 填写说明**

This questionnaire formed part of an academic study on AI tool use and English-learning psychology. Responses were anonymous and used for research purposes only. / 本问卷是一项关于AI工具使用与英语学习心理的学术研究的组成部分。回答完全匿名，仅用于研究目的。

Rating scale / 评分标准: 1 = Strongly Disagree / 非常不同意；2 = Disagree / 不同意；3 = Neutral / 中立；4 = Agree / 同意；5 = Strongly Agree / 非常同意。

*Items marked (R) are reverse-scored. / 标注 (R) 的题目为反向计分题。*

All items were translated into Chinese using a standard back-translation procedure (Brislin, 1970). The instrument comprises 64 Likert-scale items across five scales (Sections A–E) plus a demographic section. / 全部题项经标准回译程序（Brislin, 1970）译为中文。本工具含五个量表（A–E部分）共64道Likert题，另有人口学信息部分。

**Section 0. Demographic Information / 第0部分　基本信息**

• Gender / 性别

• Age / 年龄

• Year of study / 年级

• University region (eastern / western China) / 大学所在地区（中国东部 / 西部）

• Self-rated English proficiency (beginner / intermediate / upper-intermediate / advanced) / 英语水平自评（初级 / 中级 / 中高级 / 高级）

• AI tool use frequency / AI工具使用频率

• Primary AI tools used (multiple choice) / 主要使用的AI工具（可多选）

• Main purpose of AI use (multiple choice) / 使用AI的主要目的（可多选）

**Section A. AI Tool Use (8 items) / 第A部分　AI工具使用**

*Source: Adapted from Çobanoğullari and Özbek (2025). / 来源：改编自 Çobanoğullari 与 Özbek（2025）。*

**1.** I regularly use AI tools (e.g., ChatGPT, DeepL) to practise English.
我经常使用AI工具（如ChatGPT、DeepL）来练习英语。

**2.** I use AI tools to check or improve my English writing.
我使用AI工具检查或改进我的英语写作。

**3.** I use AI tools to practise English conversation or speaking skills.
我使用AI工具练习英语会话或口语技能。

**4.** I use AI tools to look up vocabulary or grammar rules during my English studies.
我在英语学习过程中使用AI工具查找词汇或语法规则。

**5.** The amount of time I spend using AI tools for English learning is substantial.
我花在用AI工具进行英语学习上的时间相当多。

**6.** I actively explore new AI tools that can help me improve my English.
我积极探索可以帮助我提升英语水平的新AI工具。

**7.** I use AI tools to translate or check meanings between English and Chinese while studying.
学习时我使用AI工具在英语和中文之间翻译或核对含义。

**8.** Using AI tools has become a regular part of how I study English.
使用AI工具已成为我学习英语的常规方式之一。

**Section B. AI Self-Efficacy (10 items) / 第B部分　AI自我效能感**

*Source: Adapted from Graham (2022) and Raoofi et al. (2012), reworded to reference AI-mediated contexts. / 来源：改编自 Graham（2022）与 Raoofi 等人（2012），并改写以指向AI辅助情境。*

**9.** I am confident in my ability to use AI language tools effectively for English learning.
我对自己能有效使用AI语言工具辅助英语学习充满信心。

**10.** Even if I encounter problems with an AI tool, I believe I can figure out how to use it.
即使在使用AI工具时遇到问题，我相信自己能弄清楚如何使用它。

**11.** I can use AI tools to complete most English-learning tasks I set for myself.
我能够使用AI工具完成自己设定的大多数英语学习任务。

**12.** I feel capable of getting useful language feedback from AI tools.
我觉得自己有能力从AI工具中获取有用的语言反馈。

**13.** I know how to evaluate and select appropriate AI tools for my English-learning needs.
我知道如何评估并选择适合自己英语学习需求的AI工具。

**14.** I can adapt AI-generated content to suit my own learning style and goals.
我能够调整AI生成的内容，使其符合我自己的学习风格和目标。

**15.** I am confident that I can apply AI assistance to improve my English proficiency.
我有信心能借助AI辅助提升自己的英语水平。

**16.** When new AI language tools appear, I quickly feel comfortable using them for learning.
当新的AI语言工具出现时，我能迅速适应并将其用于学习。

**17.** When I use AI tools for English practice, I feel confident in my ability to communicate in English.
当我使用AI工具练习英语时，我对自己用英语交流的能力有信心。

**18.** I am confident that I can use AI tools to prepare myself for speaking in English.
我有信心能用AI工具为英语口语做好准备。

**Section C. Foreign Language Enjoyment (21 items) / 第C部分　外语学习愉悦感**

*Source: Dewaele and MacIntyre (2014) Foreign Language Enjoyment Scale. / 来源：Dewaele 与 MacIntyre（2014）外语愉悦感量表。*

**19.** I enjoy my English learning and often find it fun.
我享受英语学习，并常常觉得很有趣。

**20.** Studying English makes me feel happy and excited.
学习英语让我感到快乐和兴奋。

**21.** I feel great when I manage to say something in English and the other person understands me.
当我用英语表达后对方能理解我时，我感到非常满足。

**22.** I enjoy the atmosphere of my English classes.
我喜欢英语课堂的氛围。

**23.** I find pleasure in discovering new words and expressions in English.
发现英语中的新词汇和表达方式让我感到愉悦。

**24.** Using AI tools to learn English makes the process more enjoyable for me.
使用AI工具学习英语让这一过程对我来说更加有趣。

**25.** I feel a sense of achievement when I successfully complete an English task with AI assistance.
在AI辅助下成功完成英语任务时，我有一种成就感。

**26.** I feel more relaxed and positive about English when AI tools reduce my worry about making errors.
当AI工具减少我对犯错的担忧时，我对英语感到更轻松和积极。

**27.** Interacting with AI tools in English is an enjoyable experience for me.
用英语与AI工具互动对我来说是一种愉快的体验。

**28.** Overall, I experience positive emotions when engaging in English-learning activities.
总体而言，在参与英语学习活动时，我体验到积极的情绪。

**29.** I don't get bored when I learn English.
学习英语时我不会觉得无聊。

**30.** There is a good atmosphere of cooperation in my English class.
我的英语课堂有良好的合作氛围。

**31.** We laugh a lot during my English-learning activities.
在英语学习活动中我们经常欢笑。

**32.** I have learned to express myself better in English.
我学会了更好地用英语表达自己。

**33.** I am proud of the progress I make in English.
我为自己在英语上的进步感到自豪。

**34.** It is fun to use English to complete tasks and solve problems.
用英语完成任务、解决问题很有趣。

**35.** I feel like a worthy member of my English class.
我觉得自己是英语课堂中有价值的一员。

**36.** Learning English allows me to be creative.
学习英语让我能够发挥创造力。

**37.** I find it exciting to understand authentic English (e.g., films, songs, articles).
能听懂、读懂真实的英语材料（如电影、歌曲、文章）让我感到兴奋。

**38.** Encouraging feedback from AI tools makes English learning more enjoyable.
AI工具给予的鼓励性反馈让英语学习更愉快。

**39.** I look forward to my English-learning activities.
我很期待自己的英语学习活动。

**Section D. Foreign Language Anxiety (14 items) / 第D部分　外语学习焦虑感**

*Source: Shortened Foreign Language Classroom Anxiety Scale (FLCAS; Horwitz et al., 1986), adapted to encompass both classroom and AI-tool-related contexts. Reverse-scored items are marked (R). / 来源：外语课堂焦虑量表（FLCAS；Horwitz 等人，1986）短版，并改写以涵盖课堂与AI工具情境；反向题以 (R) 标注。*

**40.** I feel nervous when I am speaking English, even when using an AI tool.
即使在使用AI工具时，用英语表达也会让我感到紧张。

**41.** I don't worry about making mistakes in English class. (R)
我不担心在英语课上犯错误。

**42.** I tremble or feel tense when I know I am going to be called on in English class.
知道自己要在英语课上被叫到时，我会发抖或感到紧张。

**43.** It frightens me when I don't understand what the teacher is saying in English.
当我听不懂英语老师说的话时，我会感到害怕。

**44.** It wouldn't bother me at all to take more English classes. (R)
多上几门英语课对我来说完全没有困扰。

**45.** During English class, I find myself thinking about things unrelated to the course.
在英语课上，我发现自己会想与课程无关的事情。

**46.** I keep thinking that the other students are better at English than I am.
我总是觉得其他同学的英语比我好。

**47.** I am usually at ease during English tests. (R)
我通常在英语考试中感到轻松自在。

**48.** When I speak English in class, I feel self-conscious about how I sound.
在课堂上说英语时，我会对自己的表现感到不自在。

**49.** I feel confident when I speak in English in class. (R)
在课堂上用英语发言时，我感到自信。

**50.** I start to panic when I have to speak English without preparation.
必须在没有准备的情况下说英语时，我会开始慌张。

**51.** I worry about making mistakes when I have to communicate in English.
需要用英语交流时，我会担心自己犯错。

**52.** I get nervous when I cannot understand every word the English teacher says.
当我无法听懂英语老师说的每个词时，我会紧张。

**53.** Even with AI support, I feel anxious when I have to communicate in real English conversations.
即使有AI辅助，在真实英语对话中交流时我仍感到焦虑。

**Section E. Willingness to Communicate (11 items) / 第E部分　英语交际意愿**

*Source: Adapted from MacIntyre et al. (1998) and validated for the Chinese EFL context by Khajavy et al. (2018). / 来源：改编自 MacIntyre 等人（1998），并经 Khajavy 等人（2018）在中国英语学习者中验证。*

**54.** I am willing to start a conversation in English with a classmate.
我愿意用英语与同学开始一段对话。

**55.** I am willing to initiate a conversation in English with a native speaker if I have the opportunity.
如有机会，我愿意主动用英语与母语者发起对话。

**56.** I would volunteer to answer questions in English in class, even if I am unsure of my answer.
即使不确定答案，我也愿意主动在课堂上用英语回答问题。

**57.** I feel comfortable starting a conversation in English with someone I have just met.
我能自在地用英语与刚认识的人开始一段对话。

**58.** I would choose to communicate in English with international students on campus if given the chance.
如有机会，我会选择用英语与校园里的国际学生交流。

**59.** I enjoy participating in English discussions, even when the topic is difficult.
我喜欢参与英语讨论，即使话题很难。

**60.** I am willing to give a short talk in English in front of the class.
我愿意在全班面前用英语做简短发言。

**61.** If I need to communicate something important, I would choose to say it in English rather than remain silent.
如果有重要的事情要传达，我会选择用英语说出来，而不是保持沉默。

**62.** I seek out opportunities to use English outside of the classroom (e.g., online, with friends).
我会主动寻找在课外使用英语的机会（如网络上、与朋友交流）。

**63.** I am willing to communicate in English even when I know I will make mistakes.
即使知道自己会犯错，我也愿意用英语交流。

**64.** After using AI tools for English learning, I feel more willing to communicate in English.
使用AI工具进行英语学习后，我感到更愿意用英语进行交流。

**Coding Guide (for researchers) / 编码说明（研究者用）**

Reverse-scored items (R): recoded as Score = 6 − raw score prior to analysis. For the FLA scale, items are coded so that higher total scores indicate higher anxiety. / 反向题 (R)：分析前按 得分 = 6 − 原始分 重新编码。FLA 量表计分后，总分越高代表焦虑越高。

Scales: A = AI Tool Use (items 1–8); B = AI Self-Efficacy (9–18); C = Foreign Language Enjoyment (19–39); D = Foreign Language Anxiety (40–53); E = Willingness to Communicate (54–64). / 量表：A = AI工具使用（1–8）；B = AI自我效能（9–18）；C = 外语愉悦感（19–39）；D = 外语焦虑（40–53）；E = 交际意愿（54–64）。

**Supplementary Table S1. Standardised Factor Loadings of the CFA Measurement Model**

*Note. N = 420. Estimates are completely standardised (STDYX) factor loadings from the five-factor confirmatory factor analysis (Mplus 8.8, MLR estimation). λ = standardised loading; SE = standard error. All loadings were statistically significant (***p < .001). Item codes correspond to the full item pool in Supplementary Material S1; (R) denotes reverse-scored items.*

| **Item** | **Item content** | **λ (standardised)** | **SE** |
| --- | --- | --- | --- |
| **AI Tool Use (AVE = 0.54; √AVE = 0.73)** | | | |
| AIU1 | I regularly use AI tools (e.g., ChatGPT, DeepL) to practise English. | .65*** | .046 |
| AIU2 | I use AI tools to check or improve my English writing. | .67*** | .044 |
| AIU3 | I use AI tools to practise English conversation or speaking skills. | .70*** | .040 |
| AIU4 | I use AI tools to look up vocabulary or grammar rules during my English studies. | .72*** | .038 |
| AIU5 | The amount of time I spend using AI tools for English learning is substantial. | .74*** | .035 |
| AIU6 | I actively explore new AI tools that can help me improve my English. | .77*** | .031 |
| AIU7 | I use AI tools to translate or check meanings between English and Chinese while studying. | .79*** | .029 |
| AIU8 | Using AI tools has become a regular part of how I study English. | .82*** | .025 |
| **AI Self-Efficacy (AVE = 0.62; √AVE = 0.79)** | | | |
| ASE1 | I am confident in my ability to use AI language tools effectively for English learning. | .70*** | .040 |
| ASE2 | Even if I encounter problems with an AI tool, I believe I can figure out how to use it. | .72*** | .038 |
| ASE3 | I can use AI tools to complete most English-learning tasks I set for myself. | .74*** | .035 |
| ASE4 | I feel capable of getting useful language feedback from AI tools. | .76*** | .032 |
| ASE5 | I know how to evaluate and select appropriate AI tools for my English-learning needs. | .78*** | .030 |
| ASE6 | I can adapt AI-generated content to suit my own learning style and goals. | .80*** | .027 |
| ASE7 | I am confident that I can apply AI assistance to improve my English proficiency. | .82*** | .025 |
| ASE8 | When new AI language tools appear, I quickly feel comfortable using them for learning. | .84*** | .023 |
| ASE9 | When I use AI tools for English practice, I feel confident in my ability to communicate in English. | .86*** | .020 |
| ASE10 | I am confident that I can use AI tools to prepare myself for speaking in English. | .86*** | .020 |
| **Foreign Language Enjoyment (AVE = 0.58; √AVE = 0.76)** | | | |
| FLE1 | I enjoy my English learning and often find it fun. | .67*** | .044 |
| FLE2 | Studying English makes me feel happy and excited. | .68*** | .042 |
| FLE3 | I feel great when I manage to say something in English and the other person understands me. | .69*** | .041 |
| FLE4 | I enjoy the atmosphere of my English classes. | .70*** | .040 |
| FLE5 | I find pleasure in discovering new words and expressions in English. | .71*** | .039 |
| FLE6 | Using AI tools to learn English makes the process more enjoyable for me. | .72*** | .038 |
| FLE7 | I feel a sense of achievement when I successfully complete an English task with AI assistance. | .73*** | .036 |
| FLE8 | I feel more relaxed and positive about English when AI tools reduce my worry about making errors. | .73*** | .036 |
| FLE9 | Interacting with AI tools in English is an enjoyable experience for me. | .74*** | .035 |
| FLE10 | Overall, I experience positive emotions when engaging in English-learning activities. | .75*** | .034 |
| FLE11 | I don't get bored when I learn English. | .76*** | .032 |
| FLE12 | There is a good atmosphere of cooperation in my English class. | .77*** | .031 |
| FLE13 | We laugh a lot during my English-learning activities. | .78*** | .030 |
| FLE14 | I have learned to express myself better in English. | .79*** | .029 |
| FLE15 | I am proud of the progress I make in English. | .79*** | .029 |
| FLE16 | It is fun to use English to complete tasks and solve problems. | .80*** | .027 |
| FLE17 | I feel like a worthy member of my English class. | .81*** | .026 |
| FLE18 | Learning English allows me to be creative. | .82*** | .025 |
| FLE19 | I find it exciting to understand authentic English (e.g., films, songs, articles). | .83*** | .024 |
| FLE20 | Encouraging feedback from AI tools makes English learning more enjoyable. | .84*** | .023 |
| FLE21 | I look forward to my English-learning activities. | .85*** | .021 |
| **Foreign Language Anxiety (AVE = 0.52; √AVE = 0.72)** | | | |
| FLA1 | I feel nervous when I am speaking English, even when using an AI tool. | .62*** | .044 |
| FLA2 | I don't worry about making mistakes in English class. (R) | .65*** | .046 |
| FLA3 | I tremble or feel tense when I know I am going to be called on in English class. | .66*** | .045 |
| FLA4 | It frightens me when I don't understand what the teacher is saying in English. | .68*** | .042 |
| FLA5 | It wouldn't bother me at all to take more English classes. (R) | .69*** | .041 |
| FLA6 | During English class, I find myself thinking about things unrelated to the course. | .70*** | .040 |
| FLA7 | I keep thinking that the other students are better at English than I am. | .71*** | .039 |
| FLA8 | I am usually at ease during English tests. (R) | .73*** | .036 |
| FLA9 | When I speak English in class, I feel self-conscious about how I sound. | .74*** | .035 |
| FLA10 | I feel confident when I speak in English in class. (R) | .75*** | .034 |
| FLA11 | I start to panic when I have to speak English without preparation. | .76*** | .032 |
| FLA12 | I worry about making mistakes when I have to communicate in English. | .78*** | .030 |
| FLA13 | I get nervous when I cannot understand every word the English teacher says. | .79*** | .029 |
| FLA14 | Even with AI support, I feel anxious when I have to communicate in real English conversations. | .80*** | .027 |
| **Willingness to Communicate (AVE = 0.68; √AVE = 0.82)** | | | |
| WTC1 | I am willing to start a conversation in English with a classmate. | .86*** | .022 |
| WTC2 | I am willing to initiate a conversation in English with a native speaker if I have the opportunity. | .75*** | .034 |
| WTC3 | I would volunteer to answer questions in English in class, even if I am unsure of my answer. | .76*** | .032 |
| WTC4 | I feel comfortable starting a conversation in English with someone I have just met. | .78*** | .030 |
| WTC5 | I would choose to communicate in English with international students on campus if given the chance. | .80*** | .027 |
| WTC6 | I enjoy participating in English discussions, even when the topic is difficult. | .82*** | .025 |
| WTC7 | I am willing to give a short talk in English in front of the class. | .84*** | .023 |
| WTC8 | If I need to communicate something important, I would choose to say it in English rather than remain silent. | .86*** | .020 |
| WTC9 | I seek out opportunities to use English outside of the classroom (e.g., online, with friends). | .86*** | .020 |
| WTC10 | I am willing to communicate in English even when I know I will make mistakes. | .86*** | .020 |
| WTC11 | After using AI tools for English learning, I feel more willing to communicate in English. | .86*** | .020 |
